# Whose Diet Suffers When Social Ties Change? Inter-Related Socioeconomic and Gender Inequalities in the Canadian Longitudinal Study on Aging Cohort

**DOI:** 10.3390/nu18203416

**Published:** 2026-10-18

**Authors:** Sanaz Mehranfar, Heather Keller, Rachel A. Murphy, Tamara R. Cohen, Gilciane Ceolin, Rana Madani Civi, Annalijn I. Conklin

**Affiliations:** 1 Faculty of Land and Food Systems, The University of British Columbia, Vancouver, BC V6T 1Z4, Canada; sanazmr@student.ubc.ca (S.M.);; 2 Department of Kinesiology and Health Sciences, University of Waterloo, Waterloo, ON N2L 3G1, Canada; 3 Schlegel-UW Research Institute for Aging, Waterloo, ON N2J 0E2, Canada; 4 Population Health Sciences, BC Cancer, Vancouver, BC V5Z 1L3, Canada; 5 School of Population and Public Health, The University of British Columbia, Vancouver, BC V6T 1Z3, Canada; 6 BC Children’s Hospital Research Institute, BC Children’s Hospital, Vancouver, BC V6H 3N1, Canada; 7 Faculty of Pharmaceutical Sciences, The University of British Columbia, Vancouver, BC V6T 1Z3, Canada; 8 Centre for Advancing Health Outcomes, Providence Health Care Research Institute, St. Paul’s Hospital, Vancouver, BC V6Z 1Y6, Canada; 9 Edwin S.H. Leong Centre for Healthy Aging, Faculty of Medicine, The University of British Columbia, Vancouver, BC V6T 1Z3, Canada

**Keywords:** socioeconomic status, social participation, fruits and vegetables, gender, prospective, CLSA

## Abstract

**Background:** Prior cohort studies have established average associations between social ties and dietary intake among aging adults, but socioeconomic status (SES) and gender differences remain unclear. This investigation of SES modification identifies those most nutritionally vulnerable to changes in social engagement. **Methods:** This prospective study of three waves of the Canadian Longitudinal Study on Aging (CLSA) included 11,900 adults aged ≥45 years. Transitions in social isolation and diversity of social participation were assessed at Waves 1 and 2, and daily fruit and vegetable intake was assessed at Waves 1 and 3. SES was measured by education and income. Random-coefficient logistic regression estimated gender-specific associations by SES. **Results:** Overall group differences were evident only for income for associations of both exposures with loss of daily fruit intake among women. High-income women remaining less socially diverse had 43% higher odds of reduced vegetable intake, while high-educated women and men had 31% and 26% higher odds, respectively. Remaining less socially diverse was associated with reduced fruit intake among women across income groups, high-educated women, and men regardless of SES. Becoming less socially diverse was associated with reduced fruit intake among high-income and high-educated women but low-SES men. Remaining socially isolated was associated with reduced fruit intake among high-income (OR 7.59 [95% CI: 3.16, 18.19]) and low-income women (1.91 [1.06, 3.45]), high-educated women (3.09 [1.70, 5.61]), and low-income men (1.76 [1.01, 3.09]). Becoming non-isolated was associated with reduced fruit intake among high-SES women. **Conclusions:** Dietary benefits of social engagement are unevenly distributed, with income as a possible moderator. Tailored strategies may better target nutritionally vulnerable groups.

## 1. Background

Promoting healthy aging is a major public health priority both globally and in Canada, given that population demographics are shifting to older ages [1,2]. Diet quality is a key modifiable determinant of health in later life, and daily intake of fruit and vegetables (FV)—a strong marker of diet quality—has been shown to reduce the risks of chronic diseases, functional decline and mortality, and thereby to support healthy aging [3,4]. Despite the known benefits of daily FV intake, suboptimal FV consumption remains common among middle-aged and older adults [5]. In Canada, more than half of older adults did not meet the recommended FV intake of five or more times per day, and intakes showed notable variation by gender and key socio-demographic characteristics [6]. Diet is shaped by multiple social and economic factors [7], referred to as the Social Determinants of Health. Social ties, in particular, are increasingly recognized as important influences on health within a public health framework [8]. They can promote healthier dietary intake through mechanisms of social control, social interaction, and social support [9]. Among the broad set of psychosocial factors contributing to health outcomes in older adults, social isolation deserves attention as a determinant of diet and healthy aging [10], especially given that 25% of older Canadians felt isolated some of the time in 2024 [11].

Social isolation is consistently associated with lower FV intake and poor diet quality [12,13,14], while greater engagement in social and leisure activities is linked to better health and higher diet quality [15,16]. Despite greater attention to the health effects of social isolation, research shows that having diverse social ties benefits health [17], particularly among women [18,19,20]. Diverse social participation may be especially important for older adults as changes in family roles or living arrangements may be compensated for by other social roles through activities such as volunteering [21], and later life transitions may require access to a wide range of social and material resources, including social support, transportation assistance, information sharing, and companionship [16]. Of significance, social isolation tends to increase across the life course, emerging in early adulthood and persisting into later life for both men and women, with steeper increases observed among women in older age [22]. Similarly, Canadian longitudinal evidence has revealed that middle-aged and older women and men differ in trajectories of social engagement over time, including transitions in social isolation and the diversity of social participation, with women generally experiencing greater declines in the number of social activities than men [23]. Moreover, specific transitions in social isolation, or in the diversity of social participation, have distinct implications for women’s or men’s subsequent eating [24], obesity [19,25], and hypertension [20].

Social and economic resources are closely intertwined in later life. Lower levels of social isolation and greater social participation are reported by individuals who also report more economic resources [26,27]. Income, education, and occupational social status (indicators of SES) are also important determinants of diet. A large body of nutrition literature demonstrates that individuals with lower SES generally have poorer dietary patterns [7,28] and that SES disparities in older adults’ diets show gender differences [29,30]. Thus, the influence of dynamic changes in social isolation and participation on dietary intake, specifically FV intake, may differ not only by gender—due to differences in how men and women experience and respond to social connections across the life course [22,29]—but may also vary among aging women or aging men by SES—due to differences in access to material resources and the size of social networks based on social roles and position [31]. Scope exists to determine whether women or men who become or stay socially diverse or those who become or stay socially isolated show a loss in daily fruit or vegetable(F/V) intake differentially according to their SES group.

This prospective study examined the potential effect modification by SES of the association of transitions in social isolation and in breadth of social participation with changes in daily F/V consumption, as a component of healthy eating, in Canadian middle-aged and older adults. We hypothesized that the association between loss or persistent lack of social engagement and loss of daily F/V intake among aging women and men would be stronger among those with lower SES.

## 2. Methods

### 2.1. Study Population

We used data from three waves of the Canadian Longitudinal Study on Aging (CLSA) Comprehensive Cohort: Wave 1 (2011–2015), Wave 2 (2015–2018), and Wave 3 (2018–2021). The CLSA Comprehensive Cohort includes 30,097 predominantly white, non-institutionalized, community-dwelling middle-aged and older adults (aged 45 and older) selected through a stratified random sampling design [32] (details in Supplemental File). We excluded participants who did not report consuming F/V at least once per day at baseline, so our outcome was the loss of daily F/V intake over time. Therefore, the analytic sample was limited to individuals who reported consuming F/V at least once daily at baseline and who had complete data on exposures, outcomes, covariates, and effect modifiers across the three waves of data collection (final analytic sample was 11,900). A participant flow diagram is provided in Supplemental Figure S1. The characteristics of the total CLSA cohort and the analytic sample are presented in Supplemental Table S1, while Supplemental Table S2 compares the analytic sample to participants who were eligible-but-excluded due to missing information.

### 2.2. Changes in Fruit or Vegetable Intake

Our primary outcome was the change from daily F/V intake at Wave 1 (2011–2015) to non-daily intake at a 6-year follow-up in Wave 3 (2018–2021); reported as the loss of daily intake. Dietary intake was assessed using the CLSA Short Diet Questionnaire (SDQ), a validated 36-item non-quantitative food frequency questionnaire for older adults [33]. The SDQ includes one question on all fruits and four questions on vegetables (green vegetables, potatoes, carrots, and other vegetables). The CLSA converted responses into daily frequencies, which we dichotomized into two variables: daily versus non-daily fruit or vegetable intake (combined items). Further details on SDQ item selection, dichotomization procedures, and outlier handling are provided in the Supplemental File. Supplemental Table S3 shows our coding method for mock CLSA responses.

### 2.3. Transitions in Social Engagement (Social Isolation and Breadth of Social Participation)

We examined changes in social isolation and diversity of social participation using CLSA data on engagement in eight different types of social activities over the past 12 months. For each activity, regular participation was defined as engaging in that activity at least once per month [19,20]. The number of activities with regular participation was then summed. Social isolation was defined as regular participation in zero or one activity per month (the lowest categories were combined due to the small number of ‘zero’ observations), whereas diverse social participation was defined as regular participation in five or more activities per month (equal to the highest tertile) and less diverse participation as regular participation in less than five activities per month [23]. We created transition variables between Waves 1 and 2 for both social isolation and diversity of social participation. Detailed information on the eight activity domains and transition categories is provided in the Supplemental File. The two transition variables were weakly correlated (Cramer’s V = 0.20).

### 2.4. Socioeconomic Status (SES)

We assessed effect modification using two objective indicators of SES measured at Wave 1. Education was dichotomized as university versus non-university education. Household income was categorized into five groups (≥C$150,000; C$100,000–149,999; C$50,000–99,999; C$20,000–49,999; and <C$20,000). Because income distributions vary across age groups—reflecting expected income changes after retirement—we constructed an age-specific binary income variable: “high-income” was defined as ≥C$100,000 for participants aged 45–64 years and as ≥C$50,000 for those aged ≥65 years. We used this age-specific approach rather than equivalizing household income for household size because household size in our data was heavily concentrated at one to two persons, limiting the additional differentiation that equivalization would provide. To assess the potential influence of the age-specific categorization on our findings, we conducted additional sensitivity analyses using a single threshold for all age groups.

### 2.5. Covariables

Consistent with our previous work, we used a directed acyclic graph (DAG) (DAGitty software, version 3.0; Nijmegen, GE, The Netherlands) to define a minimum set of covariables to address potential confounding [34,35]. All included covariables were measured at Wave 1 (baseline): age, BMI, chronic conditions, sleep deprivation, provincial GDP, provincial public spending, food insecurity, and the consumer price index for F/V (Supplemental Figure S2) [24]. Study duration was calculated from the dates recroded for participant site visits and was included given the prospective study design.

### 2.6. Sex and Gender Considerations

At Wave 1, CLSA asked participants “are you male or female?”, which may reflect biological sex, gender identity, or both. At Wave 2, participants were additionally asked to report their gender identity (male, female, transgender, transman, transwoman, genderqueer, or other). Gender identity was almost perfectly correlated with the male/female variable (Cramer’s V = 0.999). Because very few non-cisgender participants were identified and excluded, the results for women and men are interpreted as reflecting cisgender identity, gender roles, gender relations, and institutionalized gender [36].

### 2.7. Statistical Analysis

Means (SD) and frequencies (%) were used to summarize sample characteristics across the transition categories of the social engagement variables and SES indicators. Descriptive analyses applied CLSA survey inflation weights to generate population-level estimates representative of Canadians aged 45 to 85 residing in the geographic areas covered by the CLSA data collection sites [37]. A series of multivariable-adjusted random-coefficient logistic regression models were estimated on the full analytic sample (*n* = 11,900), which included a three-way interaction between the exposure, SES and gender; stratum-specific estimates were obtained by re-parameterizing the reference categories of gender and SES. The statistical significance of overall effect modification by each SES variable of the associations for women and for men was evaluated using joint Wald tests of the interaction coefficients (via STATA’s ‘testparm’ command) [38]. Given the lower statistical power of interaction tests, a significance threshold of *p* < 0.10 was used to assess effect modification [39]. Analytic weights were not applied in the inferential analyses, as the CLSA does not provide sampling weights that simultaneously account for both the individual- and higher-levels (geographic units included as random effects) [40]. The final results are presented as adjusted odds ratios (ORs) with 95% confidence intervals (95% CI). Given that the data were actual observations, we followed recommendations not to adjust for multiple comparisons so as to avoid a greater risk of type II error that may obscure potentially meaningful non-null associations [41]. We broadly interpreted the findings based on the consistency and magnitude of estimates across strata among women and among men, rather than only *p*-values. All analyses were conducted using Stata/SE version 18.

## 3. Results

The study duration averaged 70 months (SD 5.38) with a sample mean age of 62 years (9.83) and 60.64% women. The demographic and health characteristics of women and men across changes in diversity of social participation and social isolation are presented by income level in Table 1 and Table 2. The proportions of women remaining socially diverse or men remaining less socially diverse differed across income groups, while no SES pattern was seen for women or men remaining not socially isolated over time. Within each income group, demographic, health, and dietary characteristics were differentially distributed across social participation and social isolation transition categories. Supplemental Tables S4 and S5 show the distribution of sample characteristics across changes in both social engagement variables by education level.

### 3.1. Income Differences in the Prospective Association of Changes in Social Engagement with Reduced F/V Intake

There was evidence of effect modification by income only for the associations of social isolation and social participation with fruit intake among women (omnibus test of overall group effect, *p* = 0.02 for each exposure). Table 3 shows that, compared to remaining socially diverse, remaining less socially diverse was associated with subsequent loss of daily vegetable intake over time among women with high income (OR 1.43 [1.15, 1.77]). Remaining less socially diverse was associated with loss of daily fruit intake among women in both the high-income (OR 1.74 [1.36, 2.21]) and low-income (OR 1.40 [1.10, 1.78]) groups and among men in both the high-income (OR 1.40 [1.12, 1.77]) and low-income (OR 1.44 [1.05, 1.97]) groups, as compared to referents. Becoming less socially diverse was associated with the loss of daily fruit intake among high-income women (OR 1.51 [1.09, 2.07]) and among low-income men (OR 1.59 [1.03, 2.46]), compared to counterparts remaining socially diverse. Notably, becoming socially diverse was associated with the subsequent loss of daily fruit intake only among women with high income (OR 1.69 [1.21, 2.37]). Compared to remaining non-isolated, staying socially isolated was associated with the loss of daily fruit intake among women from both the high-income (OR 7.59 [3.16, 18.19])—based on a small group (*n* = 21, 10 events)—and low-income (OR 1.91 [1.06, 3.45]) subgroups and among men with low income (OR 1.76 [1.01, 3.09]). Notably, becoming non-isolated was associated with the subsequent loss of daily fruit intake only among women with high income (OR 2.70 [1.53, 4.77]), compared to staying non-isolated.

In sensitivity analyses, the use of a single threshold for household income did not alter the main findings of overall income effect modification for the association between social participation transitions and loss of daily fruit intake among women (*p* for group effect = 0.007 using ≥ C$50k and *p* for group effect = 0.016 using ≥ C$100k). Moreover, there was also evidence of income effect modification for the association between social participation transitions and loss of daily vegetable intake among women using the C$100k threshold (*p* for group effect = 0.056). However, income no longer appeared to modify the association of social isolation transitions and loss of daily fruit intake among women using either the C$50k or C$100k threshold (*p* for group effect > 0.10 for both). Overall, the transition-specific associations were largely consistent across the two alternative income thresholds.

### 3.2. Educational Differences in the Prospective Association of Changes in Social Engagement with Reduced F/V Intake

There was no evidence of overall effect modification by education for the prospective association of social isolation or social participation transitions with loss of daily F/V intake among women and among men (omnibus tests of overall education group effect *p* > 0.10). As shown in Table 4, compared to staying socially diverse, remaining less socially diverse was associated with the loss of daily vegetable intake over time among the highly educated subgroups of both women (OR 1.31 [95% CI: 1.11, 1.55]) and men (OR 1.26 [1.07, 1.48]); remaining less socially diverse was also associated with the loss of daily fruit intake among women with high education (OR 1.55 [1.27, 1.88]) but among men from both the high-education (OR 1.37 [1.12, 1.67]) and low-education (OR 1.89 [1.17, 3.05]) subgroups. Becoming less socially diverse was associated with reduced fruit intake among high-educated women (OR 1.40 [1.09, 1.80]) and among low-educated men (OR 1.98 [1.03, 3.80]), compared to counterparts staying socially diverse. Compared to staying non-isolated, remaining socially isolated (OR 3.09 [1.70, 5.61]), becoming socially isolated (OR 1.53 [1.03, 2.26]), and becoming non-isolated (OR 1.81 [1.12, 2.92]) were associated with the loss of daily fruit intake only among women with high education.

## 4. Discussion

This prospective cohort study examined how socioeconomic factors act as a moderator of the critical influence of social engagement on F/V intake during the transition from mid to later life for women and men in Canada. We found that the protective benefits of social engagement were not uniformly distributed, with income in particular being an effect modifier for social factors driving changes in women’s daily fruit intake. Specifically, changes in social isolation and changes in broad social participation were associated with the loss of daily fruit intake among women in high-income groups only or more strongly than in low-income groups. Beyond the overall effect modification findings, transition-specific associations appeared relevant within particular SES subgroups of women and men, which provide complementary information on subpopulation heterogeneity. This study adds important nuance to our understanding of the social influences on diet previously observed within Canada’s aging population [24]. The novel insights of this research are relevant theoretically and practically because our findings indicate that income modifies the dietary effect of disruptions in social isolation and broad social participation among women while also highlighting some SES inequalities for transition-specific dietary effects among women and among men. Broadly, this study demonstrates that population health research benefits from an intersectional perspective that examines heterogeneity within gender and socioeconomic groups. The dietary consequences of changes in social isolation and broad social participation were not uniformly greater among lower socioeconomic groups but varied according to both gender and SES. Thus, our original hypothesis that adverse changes in social engagement would be more strongly associated with loss of daily F/V intake among individuals with lower SES was only partially supported—more so for women. Indeed, our findings challenge simplistic assumptions that socioeconomic disadvantage alone determines vulnerability and instead highlight that the health impacts of social engagement transitions arise from the intersection of multiple social positions. Most notably, the finding that women in higher socioeconomic strata experienced heightened nutritional vulnerability when social diversity declined suggests that resource abundance does not buffer against the dietary impacts of social contraction.

### 4.1. Relevance to Previous Research

Prior research has shown that greater social cohesion, higher education, and higher income are associated with higher FV intake [42] and that lower social participation and greater economic disadvantage are associated with lower FV intake among older adults [43]. Much of the social determinants of diet literature supports a socioeconomic gradient in diet whereby individuals with lower SES report poorer diet quality. Yet, nutrition studies rarely consider socioeconomic variation in the dietary effects of social engagement, particularly from the perspective of social life dynamics as well as gender. This study is the first, to our knowledge, to assess whether the negative effects of different alterations in both social isolation and diverse social participation on F/V intake vary across SES groups among aging women and among men in Canada. Our within-group heterogeneity among women and among men makes a novel contribution to the social nutritional epidemiology literature.

Limited evidence suggests that the co-occurrence of social and economic disadvantage is associated with poorer dietary outcomes [29]. Other cross-sectional evidence shows that socially isolated individuals with low SES have higher odds of unhealthful behaviours, including low FV intake, both in the general adult population (≥18 years) [44], and among adults aged 45 years and older [45]. However, cross-sectional snapshots of social and economic circumstances are not directly comparable to prospective investigations that allow for the examination of changes in social engagement over time and their impact on subsequent FV intake. Consistent with previous research, our findings suggest that social relationships and social participation may play a particularly important role in shaping women’s health and well-being [25,46,47]. A novel finding of this study was the heightened nutritional vulnerability among several SES-gender subgroups from adverse changes in social engagement (remaining and becoming less socially diverse or isolated), especially the loss of daily fruit intake among women with high income. Although we found no consistent overall SES effect modification, a number of transition-specific associations with loss of daily FV intake were observed among high-SES women and low-SES men—a pattern that is more consistent with previous evidence linking social and economic disadvantage to poorer dietary behaviours [29]. Notably, some transition categories, particularly persistent isolation among women with high income, included small numbers of participants and outcome events. Although all strata contained both outcome events and non-events, and all estimates were derived from a single model fitted to the full sample, estimates in these strata were imprecise, as reflected by the wide confidence intervals, and their magnitude may be overestimated. These findings should therefore be interpreted with caution and replicated in larger samples.

Overall, this research revealed a more complex picture than the presumed SES gradient in the associations of loss, gain or persistent lack of social engagement on F/V intake, with transition-specific associations observed across different SES groups rather than consistently concentrated among lower-SES groups. Women with higher SES may place greater emphasis on health and weight management and have greater resources to maintain healthy dietary behaviours [48]. These behaviours are often embedded within structured daily routines and supportive social contexts [49]. Consequently, loss or chronic lack of social engagement may disrupt these routines, reduce social support and social regulation of eating behaviours, and lead to stress, thereby undermining established health-promoting habits. In other words, women with higher socioeconomic resources may have more health-related routines and social structures that facilitate healthy eating and, therefore, more to lose when these social structures are disrupted. Notably, a gain in social engagement (becoming non-isolated or more socially diverse) was also associated with reduced fruit intake among women with high SES. We interpret this result to suggest that any disruption or shift in women’s wider social environment represents a change in daily routines that could be a source of stress with consequences for their healthful eating behaviours [50,51]. It is also possible that transitioning out of isolation and becoming more diverse in social and cultural activities resulted in a greater consumption of fats and sweets [52] as a substitution that in turn reduced fruit intake. Future research should explore the SES variation in the different social life transitions in relation to changes in the composition of the whole diet, which was not possible in this study.

### 4.2. Limitations and Strengths

Self-reported measures of exposure and outcome variables may introduce misclassification bias. Recall errors in frequency of intake could have misclassified CLSA participants, but the bias would be non-differential and pull the estimates towards the null. Recall bias may also result from self-reported frequency of social activities over the past year; however, any misclassification will be consistent for both waves and would not likely alter the categorization of participants into distinct transitions in social isolation or social participation. Residual confounding can occur due to imperfect measurement of included covariates (e.g., health behaviours), and unmeasured confounding from factors not captured in the analysis (health decline, bereavement or a move) or in the dataset such as total energy intake, which was proxied by clinically measured BMI. Since all covariates are measured at Wave 1, the model does not capture confounders that change during the Wave 1-to-Wave 2 exposure window itself or during the subsequent Wave 2-to-Wave 3 window before outcome ascertainment—if such changes are associated with both the propensity to undergo the social tie transition and with loss of daily F/V intake, some residual confounding is possible—this is a standard limitation of any baseline covariate-adjusted cohort design rather than one specific to this study.

Participants in the analytic sample were more likely than eligible-but-excluded participants to have the highest level of education and income, which may limit generalizability to adults with lower educational attainment and lower income; additionally, although the baseline characteristics were otherwise similar, we cannot rule out selection bias if loss-to-follow-up (attrition) was related to changes in social engagement or F/V intake. Generalizability is further limited because the CLSA cohort is predominantly white (96%), cisgender (99%), and heterosexual (98%), and the findings may not directly apply to populations with different social, cultural, and policy contexts.

This study has several important strengths. First, the large sample size of 11,900 from a national aging cohort provided good statistical power to examine prospective associations across gender and SES groups. Second, the inclusion of two complementary measures of social engagement captured distinct aspects of social relationships, while two established indicators of SES allowed us to examine socioeconomic differences from multiple perspectives. Third, we used a theoretically driven and empirically supported DAG to adjust for a wide range of individual-level differences and socio-political variation across provinces, thereby strengthening the analytic robustness. Fourth, the use of mixed-effects models accounted for the hierarchical structure of the data with participants nested within provincial data collection sites. Fifth, the research also fully integrated sex- and gender-based analyses, which contributes to advancing chronic disease prevention for aging women who are a neglected population in medicine [53,54,55,56]. Sixth and finally, the study design leveraging three waves of CLSA data established temporal ordering between the social exposure and the dietary outcome to strengthen the assessment of prospective associations and support better causal inference [57]; additionally, the design also allowed us to assess changes in the exposure as different social engagement transitions rather than as a single measure typical of nutrition research on this topic.

### 4.3. Evidence Gaps and Future Research

These findings may inform the development of public health interventions for older Canadians that consider social risks alongside dietary guidelines, particularly community-based socially oriented programs that account for the specific socioeconomic contexts of aging populations. For example, programs for older adults experiencing social isolation or limited social participation could combine nutrition support with community meals, group cooking or food-preparation activities, or other recurring community-based social activities that incorporate food and nutrition knowledge. Such programs could additionally address practical barriers to healthy eating and social participation, such as food affordability and transportation. This study may also inform the evaluation of targeted social prescribing approaches that connect socially isolated older adults with these types of community-based programs centred on healthy eating. Social engagement, especially broad social participation, may be a potential intervention target for supporting the maintenance of daily F/V intake among aging adults, given the consistent association between chronic lack of diversity in social activities and non-daily F/V intake across SES-gender groups. Equity-focused dietary policies and messaging may also improve intervention relevance by considering the structural economic barriers intersecting with individual social life trajectories.

The present study sets the foundation to further establish whether modifying social participation would improve dietary intake. Future intervention research would need to determine whether such strategies can support dietary intake and reduce nutrition-related inequalities among aging adults. More research is needed to replicate these findings in more socioeconomically and gender-diverse populations and to examine overall dietary patterns, rather than F/V frequency alone. Although this study used total household income as a conventional SES indicator, there is scope to distinguish between income sources, which may be particularly relevant in later life when income may derive from employment-based pensions, retirement savings, government transfers, or investments. Future studies could also examine whether income source and stability influence the interplay between social engagement and dietary intake in mid-to-later life. Moreover, studies should also investigate mechanisms linking changes in social engagement to diet. Of note, standardized measures of social connectivity are needed to improve comparability across studies and identify which dimensions of social relationships are most relevant to dietary behaviours. Finally, possible causal inference from this research should be confirmed through replication and triangulation across study designs.

## 5. Conclusions

Income modified the prospective association of changes in social engagement and loss of daily fruit intake among aging women in this Canadian cohort. In addition, specific transitions, namely, loss and persistent lack, of social engagement were associated with reduced FV intake within specific SES groups of women (mostly high SES) and men (mostly low SES). This study’s results highlight the importance of considering within-group SES heterogeneity and demonstrate that social engagement may influence dietary behaviours differently across both socioeconomic and gender strata. The results also provide an impetus for research attention to the variety of social networks or activities as a possible social determinant of diet. The transition-specific results of SES differences in women’s and men’s greater nutritional vulnerability from disruptions to the social environment should motivate nutrition research, practice and policy to integrate intersectional frameworks that help elucidate how overlapping identities of gender, socioeconomic position and social life dynamics uniquely constrain or facilitate dietary resilience in an aging context.

## Figures and Tables

**Table 1:** Weighted sample characteristics across changes in breadth of social participation among women and men in the Canadian Longitudinal Study on Aging, by income level.

	High Income ^a^				Low Income ^a^			
	Remained Diverse	Remained Less Diverse	Became Less Diverse	Became Diverse	Remained Diverse	Remained Less Diverse	Became Less Diverse	Became Diverse
Women	*N* = 1426	*N* = 1332	*N* = 515	*N* = 425	*N* = 1071	*N* = 1506	*N* = 524	*N* = 417
Age (y), Mean (SD)	59.00 (10.34)	56.55 (9.47)	58.11 (10.44)	59.18 (9.67)	62.14 (9.69)	58.83 (9.26)	59.96 (9.63)	59.77 (8.5)
BMI (kg/m^2^), Mean (SD)	26.61 (5.03)	26.92 (5.57)	27.77 (6.04)	26.05 (5.09)	28.11 (6.38)	28.10 (6.4)	27.81 (5.78)	28.06 (5.71)
No chronic condition, *N* (%)	81 (7.9)	91 (9.1)	30 (5.3)	22 (6.1)	48 (5.2)	66 (6.5)	28 (5.1)	19 (5.1)
Not sleep deprived, *N* (%)	953 (69.7)	849 (56.3)	325 (67.5)	293 (72.1)	700 (65.6)	881 (56.0)	320 (61.1)	253 (61.8)
Non-daily fruit intake, *N* (%)	121 (8.1)	191 (18.6)	67 (13.4)	58 (12.9)	117 (10.5)	227 (13.2)	71 (17.7)	36 (9.0)
Non-daily vegetable intake, *N* (%)	185 (9.6)	232 (15.5)	72 (12.3)	55 (10.4)	200 (20.1)	318 (18.3)	98 (16.2)	80 (14.7)
Men	*N* = 1050	*N* = 1301	*N* = 425	*N* = 352	*N* = 377	*N* = 847	*N* = 177	*N* = 155
Age (y), Mean (SD)	60.35 (10.65)	58.34 (9.85)	59.54 (10.51)	59.10 (10.09)	60.61 (9.75)	58.15 (9.21)	58.39 (8.44)	60.07 (10.14)
BMI (kg/m^2^), Mean (SD)	28.31 (4.61)	27.40 (4.33)	28.19 (4.72)	27.99 (4.14)	28.93 (4.91)	27.71 (4.96)	28.34 (4.65)	28.31 (6.04)
No chronic condition, *N* (%)	75 (9.4)	97 (8.6)	28 (9.1)	27 (8.7)	34 (14.4)	64 (7.3)	12 (4.5)	16 (13.0)
Not sleep deprived, *N* (%)	717 (65.9)	853 (63.7)	266 (68.0)	249 (65.7)	241 (61.6)	529 (63.3)	114 (62.9)	105 (66.5)
Non-daily fruit intake, *N* (%)	141 (12.4)	232 (19.7)	62 (14.3)	50 (15.9)	65 (13.7)	194 (24.0)	44 (19.6)	25 (21.0)
Non-daily vegetable intake, *N* (%)	268 (21.9)	369 (30.3)	110 (20.1)	86 (21.1)	115 (25.5)	286 (27.9)	55 (28.7)	52 (29.7)

Note: Weighted percentages, means, and standard deviations were calculated using CLSA survey inflation weights. *N* values represent unweighted counts. All variables were reported at Wave 1, except for fruit and vegetable intake at Wave 3. ^a^: Income: high income—household income ≥C$100,000 for participants aged 45–64 years and ≥C$50,000 for those aged ≥65 years; low income—household income below these age-specific thresholds. The breadth of social participation was defined as ≥5 activities per month; the social participation transition variable is classified as: (1) remained diverse (no change in ≥5 activities at both waves); (2) remained less diverse (no change in <5 activities at both waves); (3) became less diverse (transition from ≥5 activities to <5 activities); and (4) became diverse (transition from <5 activities to ≥5 activities).

**Table 2:** Weighted sample characteristics across changes in social isolation among women and men in the Canadian Longitudinal Study on Aging, by income level.

	High Income ^a^				Low Income ^a^			
	Remained Non-Isolated	Remained Isolated	Became Isolated	Became Non-Isolated	Remained Non-Isolated	Remained Isolated	Became Isolated	Became Non-Isolated
Women	*N* = 3523	*N* = 21	*N* = 89	*N* = 65	*N* = 3177	*N* = 67	*N* = 172	*N* = 102
Age (y), Mean (SD)	57.59 (9.77)	67.47 (12.75)	62.13 (13.02)	56.43 (8.52)	60.02 (9.44)	61.88 (9.7)	60.20 (10.26)	56.34 (7.1)
BMI (kg/m^2^), Mean (SD)	26.84 (5.36)	24.89 (4.04)	26.88 (7.33)	28.54 (6.22)	28.05 (6.2)	27.95 (5.65)	28.70 (6.61)	27.42 (6.67)
No chronic condition, *N* (%)	214 (8.0)	X	X	X	147 (5.2)	X	X	6 (24.2)
Not sleep deprived, *N* (%)	2318 (64.5)	12 (67.1)	52 (50.8)	38 (49.1)	1989 (62.0)	35 (34.7)	77 (42.3)	53 (51.0)
Non-daily fruit intake, *N* (%)	396 (12.7)	10 (59.7)	14 (22.9)	17 (34.9)	393 (12.9)	15 (14.6)	27 (14.0)	16 (6.8)
Non-daily vegetable intake, *N* (%)	512 (12.3)	X	18 (26.2)	12 (14.4)	616 (17.7)	18 (24.1)	35 (21.1)	27 (18.0)
Men	*N* = 2914	*N* = 46	*N* = 97	*N* = 71	*N* = 1327	*N* = 63	*N* = 91	*N* = 75
Age (y), Mean (SD)	59.08 (10.26)	66.21 (9.45)	57.47 (8.5)	61.19 (10.77)	58.69 (9.31)	60.28 (7.72)	59.28 (8.52)	59.84 (11.55)
BMI (kg/m^2^), Mean (SD)	27.78 (4.36)	29.41 (5.77)	28.32 (6.37)	27.75 (3.44)	28.02 (4.86)	27.62 (5.48)	27.61 (5.21)	29.70 (6.48)
No chronic condition, *N* (%)	209 (8.9)	X	10 (9.4)	7 (10.5)	110 (9.9)	X	9 (5.8)	X
Not sleep deprived, *N* (%)	1955 (65.2)	26 (66.0)	61 (64.8)	43 (60.4)	846 (62.6)	34 (63.0)	62 (68.2)	47 (67.2)
Non-daily fruit intake, *N* (%)	442 (15.6)	6 (7.8)	22 (34.7)	15 (22.8)	270 (20.7)	19 (20.5)	20 (21.7)	19 (25.7)
Non-daily vegetable intake, N (%)	765 (24.8)	12 (23.1)	32 (35.9)	24 (39.3)	429 (28.7)	25 (17.1)	27 (25.2)	27 (22.8)

Note: Weighted percentages, means, and standard deviations were calculated using CLSA survey inflation weights. *N* values represent unweighted counts. All variables were reported at Wave 1, except for fruit and vegetable intake at Wave 3. X represents cells with less than 6 observations. ^a^: Income: high income—household income ≥C$100,000 for participants aged 45–64 years and ≥C$50,000 for those aged ≥65 years; low income—household income below these age-specific thresholds. Social isolation was defined as zero or one activity per month; The social isolation transition variable is classified as: (1) remained non-isolated (no change in ≥2 activities at both waves); (2) remained isolated (no change in <2 activities at both waves); (3) became isolated (transition from ≥2 activities to <2 activities); and (4) became non-isolated (transition from <2 activities to ≥2 activities).

**Table 3:** Gender-specific odds ratios (95% CIs) of non-daily fruit or vegetable intake associated with changes in social ties among women and men who were daily consumers in the Canadian Longitudinal Study on Aging (2011–2021), by income level.

	Women			Men		
	High Income ^a^	Low Income ^a^	*p*-Interaction ^†^	High Income ^a^	Low Income ^a^	*p*-Interaction
Transitions in breadth of social participation						
Non-daily vegetable intake						
Remained diverse	Ref	Ref		Ref	Ref	
Remained less diverse	1.43 ** [1.15, 1.77]	1.15 [0.94, 1.40]	>0.10	1.20 [0.998, 1.45]	1.18 [0.91, 1.55]	>0.10
Became less diverse	1.08 [0.80, 1.45]	1.00 [0.77, 1.31]	>0.10	1.02 [0.79, 1.33]	1.06 [0.72, 1.58]	>0.10
Became diverse	1.00 [0.72, 1.38]	1.02 [0.76, 1.37]	>0.10	0.96 [0.72, 1.28]	1.10 [0.74, 1.66]	>0.10
Non-daily fruit intake						
Remained diverse	Ref	Ref		Ref	Ref	
Remained less diverse	1.74 *** [1.36, 2.21]	1.40 ** [1.10, 1.78]	>0.10	1.40 ** [1.12, 1.77]	1.44 * [1.05, 1.97]	>0.10
Became less diverse	1.51 * [1.09, 2.07]	1.25 [0.91, 1.72]	>0.10	1.09 [0.79, 1.51]	1.59 * [1.03, 2.46]	>0.10
Became diverse	1.69 ** [1.21, 2.37]	0.75 [0.51, 1.11]	0.0021	1.07 [0.76, 1.52]	0.91 [0.55, 1.52]	>0.10
Transitions in social isolation						
Non-daily vegetable intake						
Remained non-isolated	Ref	Ref		Ref	Ref	
Remained isolated	0.59 [0.14, 2.55]	1.49 [0.85, 2.59]	>0.10	0.91 [0.47, 1.78]	1.37 [0.81, 2.32]	>0.10
Became isolated	1.41 [0.83, 2.39]	1.02 [0.69, 1.50]	>0.10	1.35 [0.87, 2.10]	0.85 [0.53, 1.37]	>0.10
Became non-isolated	1.27 [0.67, 2.40]	1.44 [0.91, 2.26]	>0.10	1.45 [0.87, 2.40]	1.17 [0.72, 1.92]	>0.10
Non-daily fruit intake						
Remained non-isolated	Ref	Ref		Ref	Ref	
Remained isolated	7.59 *** [3.16, 18.19]	1.91 * [1.06, 3.45]	0.010	0.83 [0.35, 1.99]	1.76 * [1.01, 3.09]	>0.10
Became isolated	1.40 [0.78, 2.51]	1.30 [0.85, 1.99]	>0.10	1.61 [0.99, 2.63]	1.09 [0.65, 1.83]	>0.10
Became non-isolated	2.70 ** [1.53, 4.77]	1.23 [0.71, 2.12]	0.0497	1.54 [0.86, 2.76]	1.31 [0.76, 2.25]	>0.10

Note: Gender-specific odds ratios (95% CIs) obtained by mixed-effects logistic regression with a 3-way interaction term (social tie × gender × income) on the sample reporting daily intake of fruits or vegetables at baseline (*n* = 11,900), conditioning on follow-up time, age, BMI, chronic conditions, sleep deprivation, provincial gross domestic product (GDP), % provincial food insecurity, provincial public spending and the fruit and vegetable consumer price index (CPI). * *p* < 0.05; ** *p* < 0.01; *** *p* < 0.001; ^†^ *p*-interaction (income × social tie) <0.10. ^a^: Income: high income—household income ≥C$100,000 for participants aged 45–64 years and ≥C$50,000 for those aged ≥65 years; low income—household income below these age-specific thresholds. The breadth of social participation was defined as ≥5 activities per month; the transition variable is classified as: (1) remained diverse (no change in ≥5 activities at both waves); (2) remained less diverse (no change in <5 activities at both waves); (3) became less diverse (transition from ≥5 activities to <5 activities); and (4) became diverse (transition from <5 activities to ≥5 activities). The social isolation transition variable is classified as: (1) remained non-isolated (no change in ≥2 activities at both waves); (2) remained isolated (no change in <2 activities at both waves); (3) became isolated (transition from ≥2 activities to <2 activities); and (4) became non-isolated (transition from <2 activities to ≥2 activities).

**Table 4:** Gender-specific odds ratios (95% CIs) of non-daily fruit or vegetable intake associated with changes in social ties among women and men who were daily consumers in the Canadian Longitudinal Study on Aging (2011–2021), by education level.

	Women			Men		
	High Education ^a^	Low Education ^a^	*p*-Interaction ^†^	High Education ^a^	Low Education ^a^	*p*-Interaction
Transitions in breadth of social participation						
Non-daily vegetable intake						
Remained diverse	Ref	Ref		Ref	Ref	
Remained less diverse	1.31 ** [1.11, 1.55]	1.07 [0.78, 1.47]	>0.10	1.26 ** [1.07, 1.48]	1.03 [0.70, 1.53]	>0.10
Became less diverse	1.01 [0.81, 1.27]	1.12 [0.73, 1.72]	>0.10	0.98 [0.78, 1.25]	1.36 [0.77, 2.38]	>0.10
Became diverse	1.13 [0.89, 1.44]	0.65 [0.39, 1.09]	0.0573	1.03 [0.80, 1.32]	0.93 [0.50, 1.72]	>0.10
Non-daily fruit intake						
Remained diverse	Ref	Ref		Ref	Ref	
Remained less diverse	1.55 *** [1.27, 1.88]	1.39 [0.95, 2.02]	>0.10	1.37 ** [1.12, 1.67]	1.89 ** [1.17, 3.05]	>0.10
Became less diverse	1.40 ** [1.09, 1.80]	1.22 [0.73, 2.03]	>0.10	1.15 [0.86, 1.52]	1.98 * [1.03, 3.80]	>0.10
Became diverse	1.32 [0.99, 1.74]	0.62 [0.32, 1.19]	0.0361	0.97 [0.71, 1.33]	1.36 [0.66, 2.81]	>0.10
Transitions in social isolation						
Non-daily vegetable intake						
Remained non-isolated	Ref	Ref		Ref	Ref	
Remained isolated	1.01 [0.49, 2.09]	1.76 [0.83, 3.70]	>0.10	1.53 [0.96, 2.44]	0.63 [0.26, 1.52]	0.08
Became isolated	1.15 [0.79, 1.67]	1.19 [0.67, 2.10]	>0.10	1.23 [0.87, 1.74]	0.68 [0.28, 1.62]	>0.10
Became non-isolated	1.23 [0.76, 1.98]	1.65 [0.91, 2.99]	>0.10	1.39 [0.92, 2.09]	1.20 [0.60, 2.37]	>0.10
Non-daily fruit intake						
Remained non-isolated	Ref	Ref		Ref	Ref	
Remained isolated	3.09 *** [1.70, 5.61]	2.15 [0.97, 4.74]	>0.10	1.42 [0.82, 2.45]	1.51 [0.65, 3.52]	>0.10
Became isolated	1.53 * [1.03, 2.26]	0.88 [0.43, 1.81]	>0.10	1.34 [0.90, 1.99]	1.66 [0.73, 3.77]	>0.10
Became non-isolated	1.81 * [1.12, 2.92]	1.42 [0.71, 2.82]	>0.10	1.50 [0.94, 2.40]	1.31 [0.62, 2.76]	>0.10

Note: Gender-specific odds ratios (95% CIs) obtained by mixed-effects logistic regression with a 3-way interaction term (social tie × gender × education) on the sample reporting daily intake of fruits or vegetables at baseline (*n* = 11,900), conditioning on follow-up time, age, BMI, chronic conditions, sleep deprivation, provincial gross domestic product (GDP), % provincial food insecurity, provincial public spending and the fruit and vegetable consumer price index (CPI). * *p* < 0.05; ** *p* < 0.01; *** *p* < 0.001; ^†^ *p*-interaction (education × social tie) <0.10. ^a^: Education: high education—university education; low education—non-university education. The breadth of social participation was defined as ≥5 activities per month; the transition variable is classified as: (1) remained diverse (no change in ≥5 activities at both waves); (2) remained less diverse (no change in <5 activities at both waves); (3) became less diverse (transition from ≥5 activities to <5 activities); and (4) became diverse (transition from <5 activities to ≥5 activities). The social isolation transition variable is classified as: (1) remained non-isolated (no change in ≥2 activities at both waves); (2) remained isolated (no change in <2 activities at both waves); (3) became isolated (transition from ≥2 activities to <2 activities); and (4) became non-isolated (transition from <2 activities to ≥2 activities).

## Data Availability

Data are available from the Canadian Longitudinal Study on Aging (www.clsa-elcv.ca) for researchers who meet the criteria for access to de-identified CLSA data.

## Supplementary Materials

The following supporting information can be downloaded at: https://www.mdpi.com/article/10.3390/nu18203416/s1, Supplemental Table S1. Comparison of the characteristics of the total Canadian Longitudinal Study on Aging (CLSA) cohort and the analytic sample. Supplemental Table S2. Comparison of participants included in the analytic sample with eligible participants excluded due to missing data. Supplemental Table S3. Coding process of mock examples of dietary data from the CLSA Short Diet Questionnaire. Supplemental Table S4: Weighted sample characteristics across changes in breadth of social participation among women and men in the Canadian Longitudinal Study on Aging, by education level. Supplemental Table S5: Weighted sample characteristics across changes in social isolation among women and men in the Canadian Longitudinal Study on Aging, by education level. Supplemental Figure S1: Flowchart of participant selection and exclusions for the analytic sample. Supplemental Figure S2: Directed acyclic graph (DAG) informing covariate selection for the association between social participation and fruit and vegetable intake. References [58,59,60] are cited in the Supplementary Materials.

## Abbreviations

BMI	Body Mass Index
CI	Confidence Interval
CLSA	Canadian Longitudinal Study on Aging
DAG	Directed Acyclic Graph
FV	Fruit and Vegetable
GDP	Gross Domestic Product
OR	Odds Ratio
SD	Standard Deviation
SDQ	Short Diet Questionnaire
SES	Socioeconomic Status
