# Supplementary material for: Whose Diet Suffers When Social Ties Change? Inter-Related Socioeconomic and Gender Inequalities in the Canadian Longitudinal Study on Aging Cohort
Source: Nutrients. 2026 Oct 18;18(20):3416. doi: 10.3390/nu18203416 (PMC13708564; doi:10.3390/nu18203416)
Supplement: Supplementary file 1 [file nutrients-18-03416-s001.zip › nutrients-4577048-supplementary.pdf]

Whose Diet Suffers When Social Ties Change? Inter-related Socioeconomic and Gender Inequalities in the Canadian Longitudinal Study on Aging Cohort

Sanaz Mehranfar, Heather Keller, Rachel A. Murphy, Tamara R. Cohen, Gilciane Ceolin, Rana Madani Civi, Annalijn I. Conklin

Table of contents

Supplemental Files-Methods

Supplemental Figure S1. Flowchart of participant selection and exclusions for the analytic sample

Supplemental Table S1. Comparison of the characteristics of the total Canadian Longitudinal Study on Aging (CLSA) cohort and the analytic sample.

Supplemental Table S2. Comparison of participants included in the analytic sample with eligible participants excluded due to missing data.

Supplemental Table S3. Coding process of mock examples of dietary data from the CLSA Short Diet Questionnaire.

Supplemental Figure S2. Directed acyclic graph (DAG) informing covariate selection for the association between social participation and fruit and vegetable intake.

Supplemental Files-Results

Supplemental Table S4: Weighted sample characteristics across changes in breadth of social participation among women and men in the Canadian Longitudinal Study on Aging, by education level.

Supplemental Table S5: Weighted sample characteristics across changes in social isolation among women and men in the Canadian Longitudinal Study on Aging, by education level.

Supplemental Methods

Study population

The Canadian Longitudinal Study on Aging (CLSA) Comprehensive Cohort includes self-reported data collected through interviews and anthropometric measurements obtained from English- or French-speaking individuals living within 25–50 km of 11 designated data collection sites. Individuals excluded from the cohort were residents of the three territories, federal First Nations reserves and other First Nations settlements in the provinces, residents of remote regions, full-time members of the Canadian Forces, and those living in institutions or with cognitive impairment at recruitment (58). To mitigate the underrepresentation of individuals with lower education and socioeconomic status in population-based research, targeted oversampling of selected areas was conducted based on census data (37). Supplemental Figure S1 presents the flowchart of participant selection, while Supplemental Tables S1 and S2 compare the characteristics of the analytic sample with those of the total Canadian Longitudinal Study on Aging (CLSA) cohort and eligible participants excluded due to missing data, respectively.

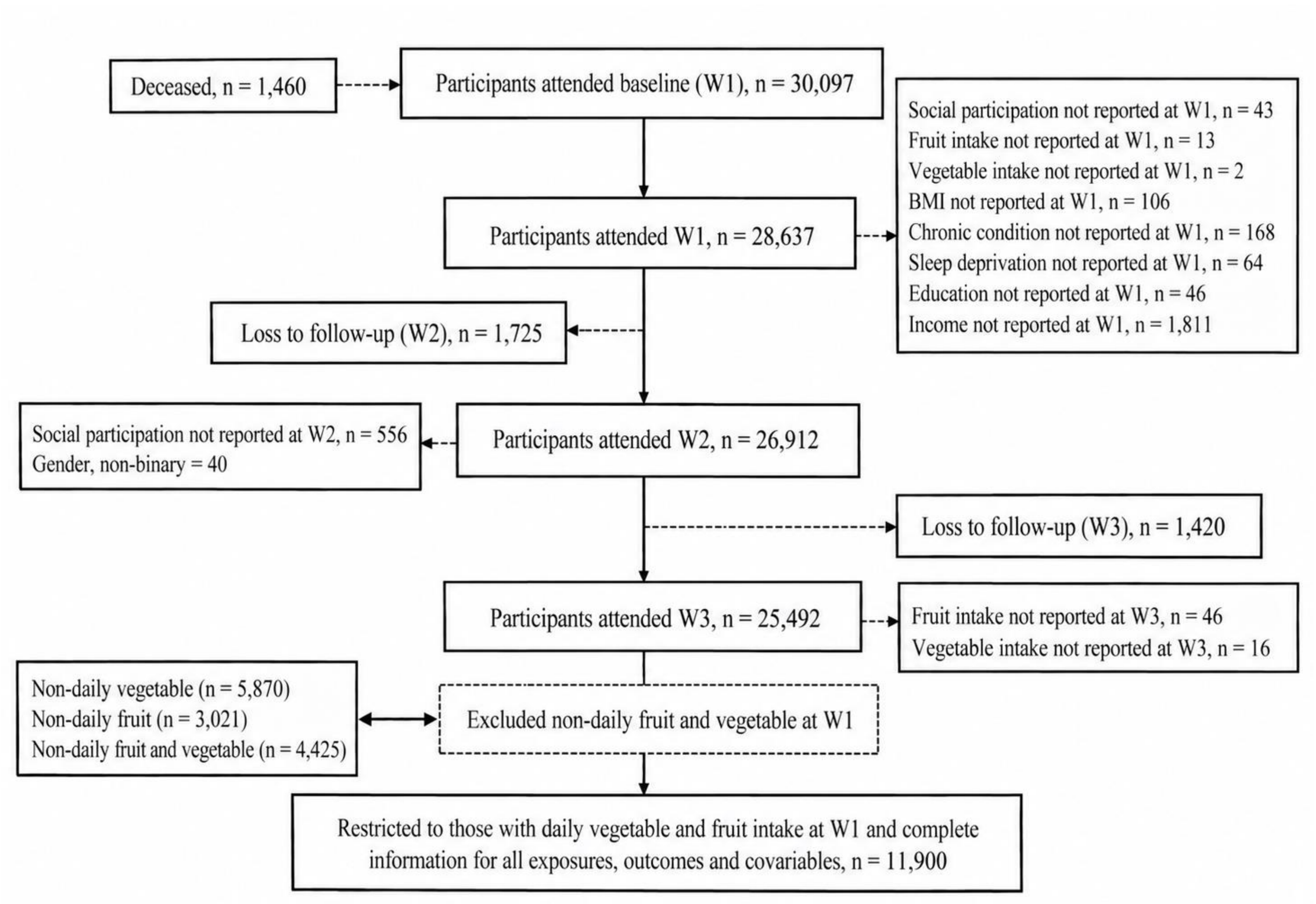

Supplemental Figure S1. Flowchart of participant selection and exclusions for the analytic sample.

Supplemental Table S1. Comparison of the characteristics of the total Canadian Longitudinal Study on Aging (CLSA) cohort and the analytic sample.

Variable	All population (n=28,637)	Analytic sample (n=11,900)
**Age (y)** (W1), Mean (SD)	62.46 (10.04)	61.98 (9.83)
**BMI (kg/m^2)** (W1), Mean (SD)	28.05 (5.43)	27.52 (5.28)
**Gender** (W2), N (%)		
Women	13,777 (48.1%)	7,216 (60.6%)
Men	13,090 (45.7%)	4,684 (39.4%)
Missing	1,770 (6.2%)	-
**Chronic condition** (W1), N (%)		
No chronic condition	1,781 (6.2%)	738 (6.2%)
At least one chronic condition	26,688 (93.1%)	11,162 (93.8%)
Missing	168 (0.59%)	
**Sleep deprivation** (W1), N (%)		
Meet at least 7H sleep	17,835 (62.4%)	7,648 (64.3%)
Not meet at least 7H sleep	10,738 (37.5%)	4,252 (35.7%)
Missing	64 (0.2%)	-
**Education** (W1), N (%)		
Post-secondary degree/diploma	22,374 (78.1%)	9,786 (82.2%)
Some post-secondary education	2,076 (7.3%)	762 (6.4%)
Secondary school graduation	2,655 (9.3%)	929 (7.8%)
Less than secondary school graduation	1,486 (5.2%)	423 (3.6%)
Missing	46 (0.16%)	-
**Household income** (W1), N (%)		
$150,000 or more	4,707 (16.4%)	2,364 (19.9%)
$100,000 or more, but less than $150,000	5,367 (18.7%)	2,524 (21.2%)
$50,000 or more, but less than $100,000	9,458 (33.0%)	4,332 (36.4%)
$20,000 or more, but less than $50,000	5,862 (20.5%)	2,248 (18.9%)
Less than $20,000	1,432 (5.0%)	432 (3.6%)
Missing	1,811 (6.3%)	0
**Change in social isolation** (W1-W2) N (%)		
Remained non-isolated	23,379 (81.6%)	10,941 (91.9%)
Remained isolated	761 (2.7%)	197 (1.7%)
Became isolated	1,287 (4.5%)	449 (3.8%)
Became non-isolated	929 (3.2%)	313 (2.6%)
Missing	2,281 (7.9%)	0
**Change in social participation** (W1-W2) N (%)		0
Remained diverse	7,513 (26.2%)	3,924 (33.0%)
Remained less diverse	12,414 (43.3%)	4,986 (41.9%)
Became less diverse	3,480 (12.2%)	1,641 (13.8%)
Became diverse	2,949 (10.3%)	1,349 (11.3%)
Missing	2,281 (7.9%)	0
**Fruit intake** (W3), N (%)		
Daily	18,135 (63.3%)	10,199 (85.7%)
Non-daily	7,311 (25.5%)	1,701 (14.3%)
Missing	3,191 (11.1%)	-
**Vegetable intake** (W3), N (%)		
Daily	16,337 (57.1%)	9,319 (78.3%)
Non-daily	9,139 (31.9%)	2,581 (21.7%)
Missing	3,161 (11.04%)	-

Supplemental Table S2. Comparison of participants included in the analytic sample with eligible participants excluded due to missing data.

	**Eligible but excluded**	**Analytic sample**
**N**	3,407 (22.3%)	11,900 (77.7%)
**Age (y)** (W1), Mean (SD)	64.903 (10.994)	61.982 (9.826)
**BMI (kg/m^2)** (W1), Mean (SD)	27.614 (5.466)	27.516 (5.276)
**Gender** (W2), N (%)		
Women	1,664 (48.8%)	7,216 (60.6%)
Men	837 (24.6%)	4,684 (39.4%)
Missing/loss to follow-up	906 (26.6%)	-
**Has at least one chronic condition** (W1), N (%)		
Has no chronic condition	193 (5.7%)	738 (6.2%)
Has at least one chronic condition	3,125 (91.7%)	11,162 (93.8%)
Missing	89 (2.6%)	-
**Sleep deprivation** (W1), N (%)		
Meet at least 7H sleep	2,134 (62.6%)	7,648 (64.3%)
Not meet at least 7H sleep	1,240 (36.4%)	4,252 (35.7%)
Missing	33 (1.0%)	-
**Household income** (W1), N (%)		
$150,000 or more	318 (9.3%)	2,364 (19.9%)
$100,000 or more, but less than $150,000	413 (12.1%)	2,524 (21.2%)
$50,000 or more, but less than $100,000	781 (22.9%)	4,332 (36.4%)
$20,000 or more, but less than $50,000	666 (19.5%)	2,248 (18.9%)
Less than $20,000	215 (6.3%)	432 (3.6%)
Missing	1,014 (29.8%)	-
**Education** (W1), N (%)		
Post-secondary degree/diploma	2,568 (75.4%)	9,786 (82.2%)
Some post-secondary education	241 (7.1%)	762 (6.4%)
Secondary school graduation, no post-secondary education	350 (10.3%)	929 (7.8%)
Less than secondary school graduation	222 (6.5%)	423 (3.6%)
Missing	26 (0.8%)	-

Changes in fruit or vegetable intake

Dietary information was collected using the 36-item Short Diet Questionnaire (SDQ), developed and implemented in the CLSA to assess usual consumption frequencies over the past 12 months for key foods and nutrients relevant to health promotion and chronic disease prevention in younger and older adults. The SDQ has been evaluated among community-dwelling older adults in a subsample of the Québec Longitudinal Study on Nutrition and Successful Aging (NuAge) (33). It has also been validated against three 24-hour dietary recalls and shown to provide reasonable estimates of usual intake frequencies for major food sources of fats, regular and low-fat foods, fibre, calcium, vitamin D, whole grains, calcium-fortified products, and fruits and vegetables (33). In the CLSA, participants reported intake frequency as daily, weekly, monthly, or yearly, followed by a question specifying the number of times within the selected period (e.g., twice per day, three times per week, once per month). All responses were converted by CLSA into times per day. We dichotomized these daily frequencies for fruits (0 = less than daily, 1 = daily), green vegetables, potatoes, carrots, and other vegetables using the same coding approach (see Supplemental Table S3). Fruit intake excluded fruit juices, consistent with Canada's Food Guide classification of fruit juice as a sweetened beverage (59). Following the National Cancer Institute Dietary Screener Questionnaire approach used in NHANES 2009–2010 (60), we excluded implausible values prior to constructing binary fruit and vegetable variables (i.e., >8 times/day for fruit; >5 times/day for green vegetables, carrots, and other vegetables; >3 times/day for potatoes). Binary variables were created at Wave 1 (to restrict the analytic sample) and at Wave 3 (to define the outcome). Although dietary guidelines recommend at least 400 grams of fruits and vegetables per day (approximately five servings), this assumption cannot be applied directly to CLSA data because the SDQ did not assess portion sizes, and participants were not provided with standard serving size definitions.

Supplemental Table S3. Coding process of mock examples of dietary data from the CLSA Short Diet Questionnaire.

Entity ID	Item	Per year/ Never	Per month	Per week	Per day	CLSA data (times per day)	Daily or non-daily
#1	Fruit			7		1	Daily
#1	Green salad				2	2	Daily
#1	Potatoes		15			0.5	Non-daily
#1	Carrots			5		0.71	Non-daily
#1	Other vegetables			14		2	Daily
#2	Fruit				5	5	Daily
#2	Green salad		25			0.83	Non-daily
#2	Potatoes			2		0.29	Non-daily
#2	Carrots	0				0	Non-daily
#2	Other vegetables			20		2.86	Daily

CLSA question was “How often do you usually eat each food group? For example, twice a day, three times a week, once a month?". CLSA converted frequency responses to times per day using 7 as the denominator for weekly intake and 30 for monthly.

Transitions in social engagement (social isolation and breadth of social participation)

We examined changes in social isolation and the breadth of social participation using CLSA data on the number of different social activities. Participants were asked how often they engaged in various activities over the past 12 months across eight domains: family, religious, sports, educational/cultural, social clubs, charity, neighborhood, and other recreational activities. We defined regular participation as taking part in an activity at least once a month or more (coded as 1), while less frequent participation was coded as 0. We then summed up the number of regularly participated activities at Waves 1 and 2 to generate a total activity score, which demonstrated acceptable internal consistency (Cronbach’s $\alpha = 0.66$). To assess social isolation, we identified participants who engaged in zero or one activity per month (combined due to the low frequency of zero scores) at each wave. To assess the breadth of social participation, we classified individuals with participation in five or more activities per month (19,20), representing the highest tertile of the activity score, as having high diversity at each wave. We then created two transition variables. The social isolation transition variable was classified as: 1) remained non-isolated (no change in

≥2 activities at both waves, reference); 2) remained isolated (no change in <2 activities at both waves); 3) became isolated (transition to <2 activities); and 4) became non-isolated (transition to ≥2 activities). The breadth of social participation transition variable was classified as: 1) remained diverse (no change in ≥5 activities at both waves, reference); 2) remained less diverse (no change in <5 activities at both waves); 3) became less diverse (transition from ≥5 activities to <5 activities); and 4) became diverse (transition from <5 activities to ≥5 activities).

Covariables

Baseline (Wave 1) covariables included study duration and (1) biological factors (age, body mass index [BMI] as a proxy for energy intake, and the presence of one or more chronic conditions including depression); (2) behavioral factor (≥7 hours of sleep per day); and (3) province-level variables (provincial GDP, provincial public spending, food insecurity, and the Consumer Price Index for fruits or vegetables (F/V), constructed based on the average recruitment date within each province. Supplemental Figure S2 presents the directed acyclic graph (DAG) used to inform covariate selection for the association between social participation and fruit and vegetable intake.

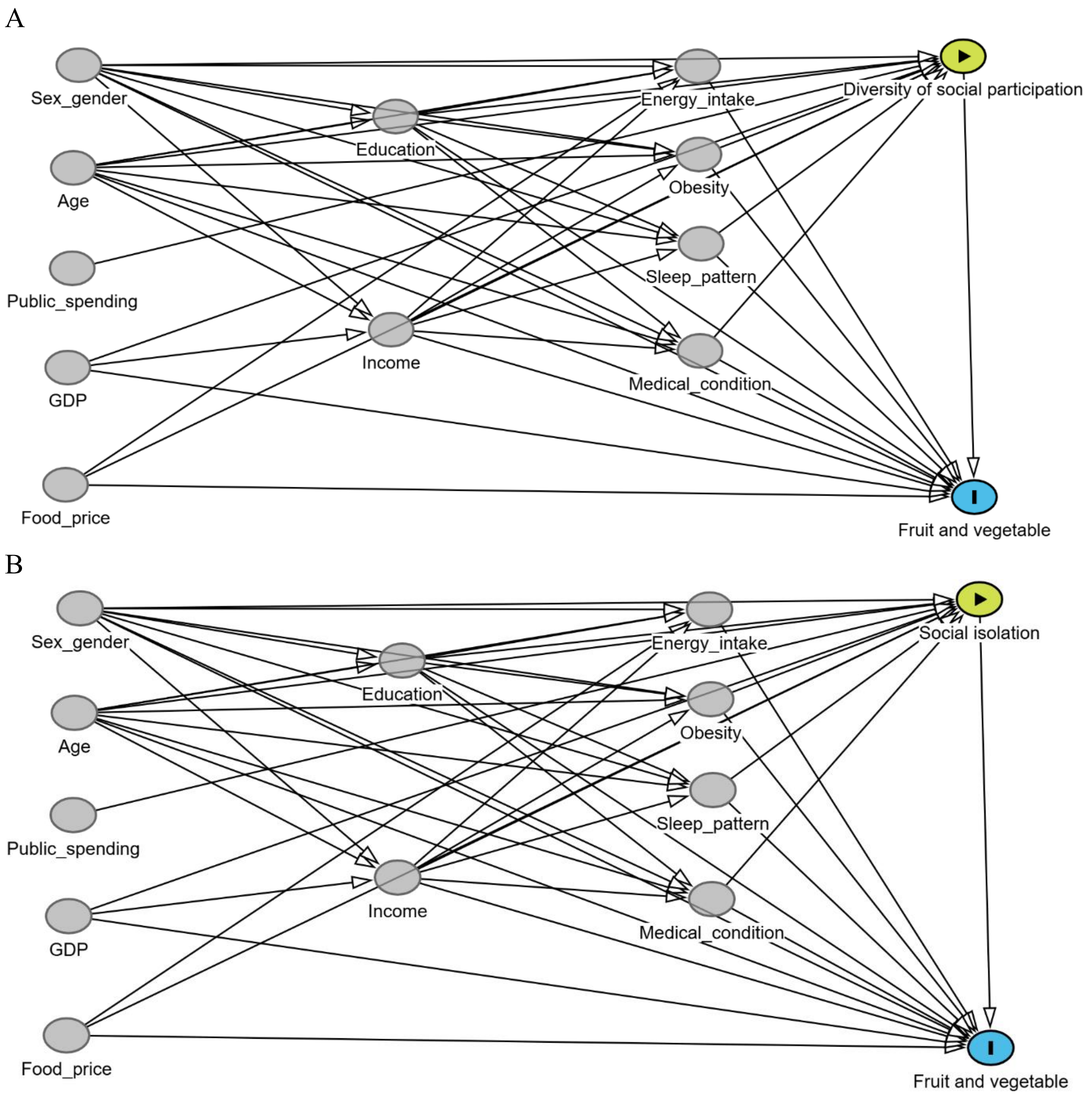

Supplemental Figure S2. Directed acyclic graph (DAG) informing covariate selection for the association between transitions in diversity of social participation (Panel A), or social isolation (Panel B), and fruit and vegetable intake. All covariates were assessed at baseline (Wave 1), while social participation transitions (isolation or diversity of social participation) were derived from Waves 1 and 2 and changes in fruit and vegetable intake were assessed between Waves 1 and 3. The exposure is the social participation transition (play symbol), and the outcome variable is the fruit and vegetable intake. The arrows represent the presumed causal association between each variable. Only arrows of confounding were retained in this DAG. GDP, Gross Domestic Product.

Supplemental Results

Supplemental Table S4: Weighted sample characteristics across changes in breadth of social participation among women and men in the Canadian Longitudinal Study on Aging, by education level.

	High education[a]				Low education[a]			
	**Remained diverse**	**Remained less diverse**	**Became less diverse**	**Became diverse**	**Remained diverse**	**Remained less diverse**	**Became less diverse**	**Became diverse**
**Women (N = 7,216)**	N = 2,146	N = 2,140	N = 848	N = 686	N = 351	N = 698	N = 191	N = 156
Age (y), mean (SD)	58.92 (9.79)	55.5 (8.45)	57.32 (9.08)	57.68 (8.48)	65.05 (9.95)	61.81 (9.72)	62.3 (10.99)	63.22 (9.13)
BMI (kg/m^2), mean (SD)	26.97 (5.81)	26.77 (5.8)	27.66 (6.23)	27.02 (5.73)	28.27 (5.35)	28.93 (6.27)	28.04 (5.27)	27.31 (5.08)
No chronic condition, N (%)	118 (7.3)	129 (8.8)	54 (7.2)	36 (6.6)	11 (4.7)	28 (5.7)	X	X
Not sleep deprived, N (%)	1,444 (70.0)	1,332 (58.9)	522 (61.2)	441 (65.7)	209 (61.2)	398 (51.3)	123 (69.6)	105 (68.7)
Non-daily fruit intake, N (%)	194 (7.9)	297 (13.3)	110 (16.0)	81 (11.7)	44 (13.2)	121 (19.9)	28 (14.9)	13 (9.0)
Non-daily vegetable intake, N (%)	311 (12.0)	392 (14.8)	126 (10.6)	112 (13.9)	74 (21.8)	158 (20.9)	44 (21.2)	23 (10.1)
**Men (N = 4,684)**	N = 1,255	N = 1,755	N = 520	N = 436	N = 172	N = 393	N = 82	N = 71
Age (y), mean (SD)	59.18 (10.01)	57.14 (8.96)	58.13 (9.26)	58.7 (9.77)	63.87 (10.59)	60.49 (10.35)	61.88 (10.71)	61.65 (10.84)
BMI (kg/m^2), mean (SD)	28.13 (4.47)	27.35 (4.63)	28.01 (4.7)	27.85 (4.57)	29.55 (5.21)	27.9 (4.57)	28.91 (4.6)	28.87 (5.64)
No chronic condition, N (%)	98 (12.0)	136 (9.7)	36 (9.5)	41 (13.2)	11 (8.1)	25 (4.9)	X	X
Not sleep deprived, N (%)	853 (64.3)	1,154 (63.5)	328 (63.6)	301 (65.4)	105 (65.1)	228 (63.6)	52 (73.0)	53 (67.7)
Non-daily fruit intake, N (%)	180 (13.3)	327 (19.5)	85 (16.0)	61 (17.9)	26 (11.5)	99 (25.8)	21 (17.3)	14 (17.0)
Non-daily vegetable intake, N (%)	329 (22.3)	531 (29.8)	135 (20.6)	117 (25.9)	54 (25.0)	124 (28.3)	30 (31.4)	21 (18.2)

NOTE: Weighted percentages, means, and standard deviations were calculated using CLSA survey inflation weights. *N* values represent unweighted counts. All variables were reported at Wave 1, except for fruit and vegetable intake at Wave 3. X represents cells with less than 6 observations. [a]: Education: high education—university education; low education—non-university education The breadth of social participation was defined as ≥5 activities per month; the transition variable is classified as: 1) remained diverse (no change in ≥5 activities at both waves); 2) remained less diverse (no change in <5 activities at both waves); 3) became less diverse (transition from ≥5 activities to <5 activities); and 4) became diverse (transition from <5 activities to ≥5 activities).

Supplemental Table S5: Weighted sample characteristics across changes in social isolation among women and men in the Canadian Longitudinal Study on Aging, by education level.

	**High education[a]**				**Low education[a]**			
	**Remained non-isolated**	**Remained isolated**	**Became isolated**	**Became non-isolated**	**Remained non-isolated**	**Remained isolated**	**Became isolated**	**Became non-isolated**
**Women (N = 7,216)**	N = 5,463	N = 55	N = 189	N = 113	N = 1,237	N = 33	N = 72	N = 54
Age (y), mean (SD)	57.1 (9.12)	58.11 (7.69)	57.31 (9.71)	54.38 (7.12)	62.59 (9.84)	66.84 (11.26)	66.61 (11.65)	58.17 (7.53)
BMI (kg/m^2), mean (SD)	26.94 (5.73)	26.7 (6.77)	28.73 (7.75)	26.45 (6.86)	28.58 (5.88)	27.42 (4.35)	26.84 (5.29)	28.96 (6.02)
No chronic condition, N (%)	319 (8.1)	X	8 (2.9)	7 (9.6)	42 (3.3)	X	X	X
Not sleep deprived, N (%)	3,542 (64.4)	32 (62.3)	97 (43.3)	68 (55.2)	765 (60.7)	15 (30.9)	32 (49.2)	23 (46.0)
Non-daily fruit intake, N (%)	612 (11.2)	16 (27.0)	32 (18.4)	22 (21.1)	177 (16.5)	9 (26.5)	9 (16.0)	11 (10.6)
Non-daily vegetable intake, N (%)	874 (13.1)	9 (11.8)	36 (16.9)	22 (12.1)	254 (19.0)	11 (23.1)	17 (32.7)	17 (21.3)
**Men (N = 4,684)**	N = 3,620	N = 80	N = 159	N = 107	N = 621	N = 29	N = 29	N = 39
Age (y), mean (SD)	57.92 (9.43)	60.73 (10.06)	57.97 (8.78)	58.19 (9.36)	61.44 (10.61)	63.97 (5.93)	58.85 (7.96)	63.09 (12.87)
BMI (kg/m^2), mean (SD)	27.56 (4.38)	28.15 (5.49)	29.13 (6.3)	29.35 (6.3)	28.63 (4.85)	28.19 (5.86)	25.32 (3.68)	28.47 (4.56)
No chronic condition, N (%)	281 (10.9)	X	17 (9.0)	10 (9.5)	38 (5.3)	X	X	X
Not sleep deprived, N (%)	2,415 (63.8)	43 (58.9)	108 (70.7)	70 (60.8)	386 (65.3)	17 (70.8)	15 (55.2)	20 (69.5)
Non-daily fruit intake, N (%)	579 (16.7)	17 (13.1)	33 (22.9)	24 (26.0)	133 (19.5)	8 (21.6)	9 (45.0)	10 (22.9)
Non-daily vegetable intake, N (%)	993 (25.6)	30 (23.5)	52 (29.7)	37 (36.7)	201 (27.7)	7 (12.5)	7 (35.9)	14 (19.3)

NOTE: Weighted percentages, means, and standard deviations were calculated using CLSA survey inflation weights. *N* values represent unweighted counts. All variables were reported at Wave 1, except for fruit and vegetable intake at Wave 3. X represents cells with less than 6 observations. [a]: Education: high education—university education; low education—non-university education. Social isolation was defined as zero or one activity per month; the social isolation transition variable is classified as: 1) remained non-isolated (no change in ≥2 activities at both waves); 2) remained isolated (no change in <2 activities at both waves); 3) became isolated (transition from ≥2 activities to <2 activities); and 4) became non-isolated (transition from <2 activities to ≥2 activities).
